# Development of a novel p72 gene-based loop-mediated isothermal amplification assay for the rapid detection of African swine fever virus in animal feed

**DOI:** 10.3389/fvets.2025.1681069

**Published:** 2025-12-15

**Authors:** Beilei Ge, Kelly J. Domesle, Janine A. Simmons, Shenia R. Young, David A. Brake, Ryan C. McDonald, Haile F. Yancy, Lindsay R. Gabbert, John G. Neilan, Chris A. Whitehouse

**Affiliations:** 1 U.S. Food and Drug Administration, Center for Veterinary Medicine, Office of Applied Science, Laurel, MD, United States; 2 SAIC, Plum Island Animal Disease Center, Greenport, NY, United States; 3 BioQuest Associates, LLC, Stowe, VT, United States; 4 U.S. Department of Homeland Security Science & Technology Directorate, Plum Island Animal Disease Center, Greenport, NY, United States

**Keywords:** African swine fever virus, animal feed, detection, loop-mediated isothermal amplification, major capsid protein

## Abstract

**Introduction:**

Rapid and reliable detection of the African swine fever virus (ASFV), the causative agent of often fatal African swine fever, in animal feed is critical for implementing timely emergency control measures.

**Methods:**

We developed a novel loop-mediated isothermal amplification (LAMP) assay targeting the ASFV p72 gene (*B646L*, encoding the major capsid protein) compatible with animal feed. Assay performance (referred to as LAMP1 in this study) was evaluated in comparison with a previously published topoisomerase II gene-based LAMP assay (LAMP2) and three p72 gene-based real-time PCR assays [two recommended by the World Organisation for Animal Health (WOAH) and the third one developed and currently used by the U.S. Department of Agriculture].

**Results:**

LAMP1 was the fastest, with positive results obtained in as early as 3.8 min [compared to at least 5.5 min for LAMP2 and 20 cycles for real-time PCRs (~15 min)]. These assays could detect ASFV from 10^−1^ to 10^2^ copies using synthetic DNA. LAMP1 detected as low as 10^1^ TCID_50_/mL of ASFV BA71V stock and had a comparable performance to the USDA real-time PCR assay using both inclusivity (36 ASFV synthetic DNAs and isolates) and exclusivity (13 porcine viruses) panels. On applying six different DNA extraction methods to a variety of animal feed sample types (e.g., complete swine feed, soybean meal), variable yet mostly limited assay inhibitions were observed. When swine feed was inoculated with the ASFV BA71V stock at 10^5.1^ TCID_50_/g, the newly developed LAMP1 assay reliably detected the virus within 7 min, in contrast to at least 20 min by the USDA real-time PCR (29 cycles).

**Discussion:**

Further validation of this novel p72 gene-based LAMP assay in animal feed will pave the way for its adoption as a rapid screening tool in ASFV feed surveillance, as well as potential application in outbreak response and recovery efforts, to safeguard the nation’s animal feed supply.

## 1 Introduction

African swine fever (ASF) is a highly contagious and often deadly hemorrhagic disease of domestic pigs and wild boars (1). A notifiable animal disease designated by the World Organisation for Animal Health (WOAH) (2), ASF is enzootic in sub-Saharan Africa, and since gaining entrance to Georgia in 2007 (3) and China in 2018 (4), it has rapidly spread to numerous other Asian and European countries with domestic and wild pig populations (1, 5). The disease has never been reported in the United States, but has recently gained a foothold in the Western Hemisphere on the island of Hispaniola (1, 6, 7). In the absence of widely available, effective ASF vaccines or treatments, this economically devastating transboundary disease continues to pose a major threat to the global pig population and agricultural economy (6–8). Effective surveillance, strict biosecurity, and rapid diagnostics are essential for preventing and controlling ASF spread worldwide (9).

The etiologic agent for ASF, African swine fever virus (ASFV), is a large, enveloped, double-stranded DNA virus and the sole member of the genus *Asfivirus* within the family *Asfarviridae* (10). Historically, at least 25 ASFV genotypes existed based on sequence analysis of the partial p72 gene (*B646L*), encoding the major capsid protein (11). Recently, a modern approach to classify the ASFV genotypes based on a thorough, sophisticated analysis of published p72 amino acid sequences reduced the total number of ASFV genotypes to only six (12). Distinct ASFV genotypes are circulating in different regions of the world, with genotypes I and II and their hybrids in Asia, Europe, and the Americas (4, 13, 14); whereas, other genotypes have only been reported on the African continent (15). Due to its relatively high environmental stability, ASFV has complex transmission routes, including direct contact with infected pigs or contaminated fomites, indirect contact by feeding pigs garbage or other infected products, or via various *Ornithodoros* spp. soft tick vectors that feed on ASFV-infected pigs (7, 9, 16). Though considered a low risk for ASFV introduction into an ASFV-free country (17, 18), animal feed (animal origin, plant origin, or supplements) can contribute to ASF disease spread once established in a country, considering the viruses’ remarkable stability in animal feed (19–21). Rapid and reliable methods for detecting ASFV in animal feed are critical for ASF surveillance, preparedness, and response, but currently, such methods are lacking.

Globally, WOAH provides guidance on ASFV diagnostic tests in susceptible pigs, such as virus isolation, PCR, and immunoassay, as detailed in the *Terrestrial Manual* (22). Methods such as those combining CRISPR/Cas12a or CRISPR/Cas13a (clustered regularly interspaced short palindromic repeats/CRISPR-associated proteins) and recombinase polymerase amplification (PRA) or recombinase-aided amplification (RAA) (targeting the p17 gene *D117L*, the p22 gene *KP177R*, the p72 gene *B646L*, or a conserved protease gene *S273R*) have been explored (23–27). Validated PCR assays (many targeting the p72 gene *B646L*) are considered excellent choices for rapid ASFV diagnostics from animal samples, as exemplified by two real-time PCR assays (28, 29). A U.S. Department of Agriculture (USDA)’s Agriculture Research Service (ARS) p72 gene-based real-time PCR assay (30), adopted and recently updated by the USDA’s Animal and Plant Health Inspection service (APHIS) (31), has been approved for ASFV detection in seven animal diagnostic sample types (whole blood, tonsil, spleen, lymph node, blood swabs, spleen pulp swabs, and dried blood cards) (personal communication with the National Animal Health Laboratory Network coordinator), and the evaluation in oral fluid was complete (32). Similar to common challenges encountered for pathogen detection in human food (33), animal feed presents as a challenging matrix for ASFV molecular detection due to assumed low viral loads, heterogeneous viral distributions, complex and refractory compositions, and the presence of assay inhibitors. Methods need to be developed that both effectively concentrate and isolate ASFV target analytes from animal feed and have a good tolerance to potential assay inhibitors in animal feed.

Loop-mediated isothermal amplification (LAMP) (34) has emerged as a powerful alternative to PCR for the rapid and reliable detection of pathogens and other contaminants in food (35, 36). The main advantages of LAMP, compared with PCR, include isothermal amplification, high tolerance to food inhibitors, detection platform flexibility, high specificity, sensitivity, and speed (37–39). LAMP is also field deployable and amenable to resource-limited areas due to its simple setup (instrument) and versatile signal monitoring approaches (40). The speed and portability of LAMP present major advantages over PCR in outbreak situations. The first ASFV LAMP assay was reported in 2010, targeting the topoisomerase II gene (*P1192R*) using blood and tissue samples (41). Subsequent field evaluation using blood swabs and serum samples during an outbreak in Timor-Leste was reported, in which the DNA extraction step was removed, and an internal amplification control (IAC) testing in separate wells was implemented (42). Additional LAMP assays targeting the p72 gene and various other ASFV genes have been reported (43, 44). To date, evaluations of these assays in animal feed have not been reported.

To address this crucial gap, the present study aimed to develop and evaluate a novel p72 gene-based LAMP assay (herein referred to as LAMP1) for ASFV rapid detection in complete animal feed and feed ingredients. The topoisomerase II gene-based LAMP (41) (herein referred to as LAMP 2) and three p72 gene-based real-time PCRs, two recommended by the WOAH (28, 29) (herein referred to as WOAH-King and WOAH-Fernandez-Pinero real-time PCRs) and the third one by the USDA (30, 31) (two versions, herein referred to as USDA-APHIS and USDA-Zsak real-time PCRs), were included for comparison. Assay performance was evaluated based on sensitivity, specificity, and applicability in animal feed (including the effects of DNA extraction methods). Both ASFV stocks and synthetic DNAs were evaluated, as well as a variety of animal feed sample types.

## 2 Materials and methods

### 2.1 ASFV synthetic DNAs and virus stocks

ASFV synthetic DNAs (*n* = 27; Table 1) were used for the initial evaluation of assay sensitivity and specificity. Twenty-six full-length ASFV p72 genes (*B646L*, 1,941 bp) representing various genotypes were downloaded from the National Center for Biotechnology Information (NCBI) and synthesized in the form of gBlocks Gene Fragments (Integrated DNA Technologies, Coralville, IA, USA). A certified reference (Quantitative Synthetic ASFV DNA, ATCC VR-3283SD; American Type Culture Collection, Manassas, VA, USA) consisting of eight ASFV gene fragments (from *B646L*, *A489*, *505-2R*, *C717R*, *B962L*, *B119L*, *G1340L*, and *D1133L*) was also used. Additionally, one partial-length topoisomerase II gene (*P1192R*, 1,969 bp; footnote in Table 1) gBlocks Gene Fragment was also used specifically for LAMP2 sensitivity evaluation.

**Table 1:** Assay specificity evaluated using ASFV synthetic DNAs and virus stocks.

ASFV sample ID^a^ or non-ASFV virus name	ASFV genotype^b^ or non-ASFV virus ID	LAMP1 T_p_ (min)	USDA-Zsak or USDA-APHIS real-time PCR C_T_ (no. of cycles)^c^	WOAH-King real-time PCR C_T_ (no. of cycles)	WOAH-Fernandez-Pinero real-time PCR C_T_ (no. of cycles)
ASFV synthetic DNAs (gBlocks Gene Fragments; *n* = 26)					
47/Ss/2008	I	5.5	26.1	23.5	24.5
Benin 97/1	I	6.2	27.1	24.2	25.3
E75	I	5.7	27.3	24.8	24.4
K49	I	6.2	25.1	22.9	24.3
L60	I	5.4	26.0	22.9	25.0
Mkuzi 1979	I	5.8	27.9	25.9	26.3
Belgium 2018/1	II	5.5	27.4	25.0	27.1
China/2018/AnhuiXCGQ	II	5.7	26.7	23.0	26.7
GEO 1/2007	II	5.0	28.9	27.8	27.9
IND/AS/SD-02/2020	II	5.4	28.8	25.8	29.2
POL/2015/Podlaskie	II	5.3	26.7	22.9	27.3
Russia/Kashino 04/13	II	5.4	26.9	23.5	26.6
Tanzania/Rukwa/2017/1	II	5.1	26.3	22.7	25.7
Pretorisuskop-96-4	III	5.6	25.3	23.1	26.5
RSA_2_2004	III	8.2	35.3	Undetermined	27.0
RSA_2_2008	III	9.0	34.7	39.9	26.2
SPEC_57	III	8.3	35.2	35.1	26.8
Tengani 62	III	5.3	26.7	23.2	25.0
Warmbaths	III	5.2	26.4	23.6	26.8
Warthog	III	5.4	26.8	23.5	24.3
Zaire	III	8.3	35.5	Undetermined	27.3
Ken06. Bus	V	8.6	26.5	23.7	27.6
R35	V	blank^d^	26.3	23.6	29.0
Malawi Lil-20/1	VI	6.7	25.1	23.2	29.3
BUR/18/Rutana	X	7.2	26.6	23.4	28.6
ETH/1	Unknown	7.8	26.7	22.6	27.0
Synthetic DNA (certified reference material; *n* = 1)					
ATCC VR-3283SD	Unknown	5.4	26.5	27.3	27.2
ASFV stocks (*n* = 9)					
BA71V	I	5.0	26.6	NA	NA
Spencer/51	I	8.9	28.5	NA	NA
Georgia	II	7.5	29.6	NA	NA
Pretoria	III	8.1	29.6	NA	NA
Tengani 62	III	8.2	28.6	NA	NA
Zaire	III	11.1	30.0	NA	NA
Kenya06. Bus	V	10.8	28.0	NA	NA
Malawi Lil-20/1	VI	8.9	28.8	NA	NA
Chiredzi/83/1	Unknown	8.5	29.8	NA	NA
Non-ASFV DNA viruses (*n* = 4)					
Porcine circovirus 2	N/A	Undetermined	Undetermined	NA	NA
Porcine cytomegalovirus	N/A	Undetermined	Undetermined	NA	NA
Porcine parvovirus	NADL-2 (ATCC VR-742)	Undetermined	Undetermined	NA	NA
Pseudorabies virus	ATCC VR-135	Undetermined	Undetermined	NA	NA
Non-ASFV RNA viruses (*n* = 9)					
Bovine vesicular disease virus (bovine viral diarrhea virus)	Singer	Undetermined	Undetermined	NA	NA
Classical swine fever virus	Brescia	Undetermined	Undetermined	NA	NA
Foot-and-mouth disease virus	A24	Undetermined	Undetermined	NA	NA
	Asia1 Shamir	Undetermined	Undetermined	NA	NA
	O1 Manisa	Undetermined	Undetermined	NA	NA
	SAT2	Undetermined	Undetermined	NA	NA
Porcine epidemic diarrhea virus	CO-025PDC-1301	Undetermined	Undetermined	NA	NA
Vesicular stomatitis virus	Indiana1	Undetermined	Undetermined	NA	NA
	New Jersey	Undetermined	Undetermined	NA	NA

a All gBlocks Gene Fragments were full-length p72 genes (1,941 bp) of various ASFV strains. Another gBlocks Gene Fragment GEO 1/2007-P1192R (not shown) was a partial-length topoisomerase II gene (*P1192R*; 1,969 bp) of the ASFV Georgia strain, targeted by LAMP2 only.

b ASFV genotypes were determined using the recently published new scheme, where the published p72 amino acid sequences were used for classification (12).

c ASFV synthetic DNAs were evaluated by the USDA-Zsak real-time PCR, whereas ASFV and non-ASFV stocks were evaluated by the USDA-APHIS real-time PCR.

d ASFV synthetic DNA R35 was detected starting at 10^6^ copies/reaction with a T_p_ of 11.9 min.

Stock solutions (10 ng/μL) of gBlocks Gene Fragments were made by resuspending the lyophilized DNA in molecular-grade water (Thermo Fisher Scientific, Waltham, MA, USA) with incubation at 50 °C for 15–20 min in a heat block, and the concentration was verified with Quant-iT Broad-Range dsDNA Assay Kit on a Qubit fluorometer (Thermo Fisher Scientific). Working stocks (1 ng/μL) were made by diluting the stock solutions in molecular-grade water, and aliquots (10 μL) of gBlocks Gene Fragments working stocks, along with aliquots (10 μL) of ATCC VR-3283SD, were stored at −20 °C before use, avoiding more than three freeze/thaw cycles.

ASFV virus stocks (*n* = 9; Table 1) were used to further evaluate assay sensitivity, inclusivity, and application in animal feed. Media and reagents were obtained from Thermo Fisher Scientific unless specified otherwise. Briefly, to produce the working virus stock of tissue culture-adapted ASFV BA71V (45), Vero cells (African green monkey kidney epithelial cells, ATCC CCL-81) were propagated in T175 flasks containing complete growth media [Dulbecco’s Modified Eagle Medium (DMEM, Gibco, USA) supplemented with 10% fetal bovine serum (FBS, R&D Systems, USA), 1% MEM non-essential amino acids (NEAA, Gibco), 1% sodium pyruvate (Gibco), and 1% antibiotic-antimycotic (A/A, Gibco)]. ASFV BA71V was incubated with a confluent monolayer of Vero cells for 5–6 days. After two freeze–thaw cycles, viral supernatants were pooled and centrifuged at 3,200 × *g* for 20 min at 8 °C. To produce working virus stocks of other ASFV isolates (Table 1), in-house isolated primary swine macrophages were seeded in T25 Primaria flasks containing macrophage growth media [RPMI-1640 with HEPES/L-Glutamine (Life Technologies, USA) supplemented with 30% L929 cell supernatant (produced in-house: briefly, supernatant from L929 cells grown over a 10- to 12-day period was harvested, 0.22 μM filtered twice, and stored frozen), 10% FBS, 1% A/A, and 1% gentamicin (Gibco)]. ASFV isolates were incubated with a monolayer of primary swine macrophages for 3 days. After two freeze–thaw cycles, viral supernatants were pooled, sonicated with Misonix Ultrasonic Liquid Processor S-4000 at full power (100 amplitude) with three 10-s bursts, and centrifuged at 3,220 × *g* for 10 min at 4 °C. All collected supernatants were aliquoted and stored at −80 °C.

### 2.2 Non-ASFV DNA and RNA porcine virus stocks

Non-ASFV DNA and RNA porcine viruses (*n* = 13; Table 1) were used to evaluate assay exclusivity. Working non-ASFV stocks were graciously provided by Dr. Wei Jia from the USDA-APHIS Reagents and Vaccine Services Section (RVSS) and Dr. Jianqiang Zhang from Iowa State University Veterinary Diagnostic Laboratory. Foot-and-mouth disease virus (FMDV) stocks were previously produced in-house (stocks were produced following the same procedure for ASFV BA71V stock, except confluent LF-BK ανβ6 cells (46) in T25 flasks were infected with the FMDV strain) and stored at −80 °C.

### 2.3 LAMP assays

The six novel primers (Table 2) for the p72 gene-based LAMP1 assay were designed using the LAMP Designer V1.16 (PREMIER Biosoft, Palo Alto, CA) against the ASFV genotype II reference genome Georgia 2007/1 (GenBank accession number FR682468.2). The primer set consisted of two outer (F3 and B3), two inner (FIP and BIP), and two loop (loopF and loopB) primers that targeted eight conserved regions of the p72 gene (*B646L*), encoding the major capsid protein. Table 2 also lists the primer set for the topoisomerase II gene-based LAMP assay (LAMP2) targeting the *P1192R* gene (41).

**Table 2:** ASFV LAMP and real-time PCR primers and probes.

Assay and primer name	Sequence (5′-3′)^a^	Position^b^	Reference
LAMP1			This study
ASFV1-F3	CCAACAATAACCACCACGA	1,337–1,355	
ASFV1-B3	TACCATGAGCAGTTACGGA	1,621–1,639	
ASFV1-FIP	CAACATGTCCGAACTTGTGCC-ACCTACCTGGAACATCTCC	1,460–1,480 (F1c) 1,413–1,431 (F2)	
ASFV1-BIP	TAACGCCATTATGCAGCCCAC-GATGAACATGCGTCTGGAA	1,482–1,502 (B1c) 1,544–1,562 (B2)	
ASFV1-loopF	AATCTCGGTGTTGATGAGGATT	1,438–1,459	
ASFV1-loopB	TCACCACGCAGAGATAAGC	1,503–-1521	
LAMP2			(41)
James-F3	GGCGCAAAATTTTAGCCGG	2,153–2,171	
James-B3	GCCGAAGCTTCCTATGCC	2,341–2,358	
James-FIP	GCAACGTAGCCCCCGAACTG-GAAATGCTTCGCYTCCAACA	2,217–2,237 (F1c) 2,178–2,197 (F2)	
James-BIP	ATCACCATGGCGACATGTCGT-GGATAGAGGTGGGAGGAGC	2,252–2,272 (B1c) 2,312–2,330 (B2)	
James-loopF	AAAAACCTTTCGTTCACGGT	2,198–2,217	
James-loopB	AAAAGCCGCCCAGTATTACC	2,289–2,308	
USDA-Zsak real-time PCR			(30)
Zsak-forward	CCTCGGCGAGCGCTTTATCAC	1,053–1,073	
Zsak-reverse	GGAAACTCATTCACCAAATCCTT	1,093–1,115	
Zsak-probe	6-FAM-CGATGCAAGCTTTAT-MGB-NFQ	1,075–1,089	
USDA-APHIS real-time PCR			(31)
ASF forward primer	CTTCGGCGAGCGCTTTATCAC	1,053–1,073	
ASF reverse primer	GGAAATTCATTCACCAAATCCTT	1,093–1,115	
ASF probe	6-FAM-CGATGCAAGCTTTAT-MGB-NFQ	1,075–1,089	
WOAH-King real-time PCR			(28)
King-forward	CTGCTCATGGTATCAATCTTATCGA	1,628–1,652	
King-reverse	GATACCACAAGATCRGCCGT	1858–1877	
King-probe	6-FAM-CCACGGGAGGAATACCAACCCAGTG-6-TAMRA	1762–1786	
WOAH-Fernandez-Pinero real-time PCR			(29)
Fernandez-Pinero-forward	CCCAGGRGATAAAATGACTG	648–667	
Fernandez-Pinero-reverse	CACTRGTTCCCTCCACCGATA	695–715	
Fernandez-Pinero-probe	6-FAM-TCCTGGCCRACCAAGTGCTT-BHQ	673–692	

a Underlined sequences are either F2 or B2 as indicated.

b The positions are numbered based on the coding sequencing of the *B646L* gene (*P1192R* for LAMP2) derived from the ASFV genotype II reference genome Georgia 2007/1 (GenBank accession number FR682468.2).

For the Fernandez-Pinero probe, the standard probe shown was used to substitute the universal probe library one in the original publication as prescribed by the *Terrestrial Manual*.

The LAMP assays were performed on two fluorescence-based platforms, a small, portable real-time fluorometer (Genie II; OptiGene Ltd., West Sussex, UK), and a standard real-time PCR instrument (7500 Real-Time PCR System; Thermo Fisher Scientific). Briefly, the LAMP reaction mix (25 μL total) consisted of 1 × GspSSD2.0 Isothermal Mastermix (ISO-004; OptiGene Ltd.), 1 × primer mix (0.1 μM each outer primer, 1.8 μM each inner primer, 1 μM each loop primer; Integrated DNA Technologies), and 5 μL of DNA template. A positive control (gBlocks Gene Fragments for the ASFV Georgia strain GEO 1/2007 or ASFV BA71V stock) and a no template control (molecular-grade water) were included. The LAMP reaction profile comprised amplification at 65 °C for 30 min, followed by an anneal/melt curve analysis between 80 °C and 98 °C (0.05 °C decrement per s in Genie II and 1.5% ramp rate in 7500). Fluorescence readings were acquired in real-time using the FAM channel. The time-to-positive results, peak ratio (T_p_; min) on Genie II or cycle threshold (C_T_; number of cycles, 1 min/cycle) on 7500, were determined when the fluorescence ratio reached the maximum value of the amplification rate curve or when the fluorescence reading crossed the threshold set in the exponential phase of the amplification curve, respectively. The annealing/melting temperatures of the LAMP amplicons, anneal peak (T_a_; °C) on Genie II or melting temperature (T_m_; °C) on 7500, were obtained when the fluorescence derivative reached the maximum value of the anneal derivative curve or the melting curve, respectively. Samples were considered LAMP-positive with T_p_/C_T_ within 20 min (i.e., 20 cycles on 7500) and a T_a_/T_m_ of approximately 86 °C, reflecting specific amplifications of target ASFV p72 (LAMP1) or topoisomerase II (LAMP2) genes.

### 2.4 Real-time PCR assays

Three p72 gene-based real-time PCR assays, WOAH-King (28), WOAH-Fernandez-Pinero (29), and USDA-Zsak (30) (primers and probes shown in Table 2), were carried out on the 7500 Fast Real-Time PCR System when ASFV synthetic DNAs were used for the initial evaluation of assay sensitivity and specificity. The harmonized real-time PCR reaction mix (25 μL total) contained 1 × TaqMan Fast Advanced Master Mix (Thermo Fisher Scientific), 1 × primer/probe mix as described in the original publications (Integrated DNA Technologies), and 5 μL of DNA template. The program consisted of initial inactivation at 95 °C for 20 s, and 45 cycles of denaturation at 95 °C for 3 s and annealing/extension at 60 °C for 30 s.

When virus stocks were used to further evaluate assay sensitivity and specificity in animal feed, the USDA-APHIS real-time PCR was carried out on the 7500 Real-time PCR System. The reaction mix (25 μL total) contained 1 × TaqManFast Virus 1-Step Master Mix (Thermo Fisher Scientific), 0.3 μM forward/reverse primers, 0.2 μM FAM-labeled probe, and incorporated an exogenous internal positive control (1 × Xeno Liz internal control reagent and 1 × Xeno RNA/DNA). The real-time PCR program on the 7500 consisted of initial inactivation at 95 °C for 20 s, and 45 cycles of denaturation at 95 °C for 10 s and annealing/extension at 60 °C for 30 s. Fluorescence readings were obtained in real-time from the FAM channel, and the C_T_ values (number of cycles) were recorded when the fluorescence level crossed the threshold value set in the amplification curve exponential phase.

### 2.5 Sensitivity and specificity

Both ASFV synthetic DNAs and virus stocks were used for evaluating assay sensitivity (limit of detection, LOD) and specificity (inclusivity and exclusivity). Sensitivity templates were independently tested (using different LAMP or real-time PCR master mixes) three times, whereas specificity templates were tested once. Briefly, the assay LODs were determined initially using 10-fold serial dilutions (ranging from 10^6^ to 10^0^ copies/reaction) of the gBlocks Gene Fragment GEO 1/2007 for the ASFV Georgia strain p72 gene (GEO 1/2007-*P1192R* for the ASFV Georgia strain topoisomerase II gene for LAMP2), ranging from 10^6^ to 10^0^ copies/reaction, and Quantitative Synthetic ASFV DNA ATCC VR-3283SD, ranging from 8.8 × 10^5^ to 0.8 copies/reaction. The Vero cell-adapted ASFV BA71V and eight ASFV isolates were used to further evaluate the assay LODs. DNA was extracted from 10-fold serially diluted virus stocks [ranging from approximately 10^7^ to 10^2^ TCID_50_/mL (50% tissue culture infectious dose/mL)] using the ChargeSwitch gDNA Rendered Meat Purification Kit (“ChargeSwitch” in short; Thermo Fisher Scientific) with 500 μL input volume.

Inclusivity was evaluated initially using 26 gBlocks Gene Fragments and ATCC VR-3283SD (Table 1) at approximately 10^4^ copies/reaction. The ASFV BA71V stock at approximately 10^4^ TCID_50_/mL and eight other ASFV isolates representing various genotypes (Table 1) at approximately 10^4^ TCID_50_/mL or HAD_50_/mL (50% hemadsorption dose/mL) were used to further evaluate the assay inclusivity. Exclusivity was evaluated using a range of non-ASFV DNA and RNA porcine virus stocks (Table 1) at approximately 10^7^ TCID_50_/mL. Nucleic acids were extracted from the virus stocks of both ASFV and non-ASFV DNA isolates similarly using ChargeSwitch. Nucleic acids from non-ASFV RNA isolates were extracted by the MagMax Pathogen RNA/DNA Kit (Thermo Fisher Scientific) using the KingFisher Flex System (Thermo Fisher Scientific).

### 2.6 Animal feed inhibitor evaluation with different DNA extraction methods

Pellets and supernatants from eight animal feed samples were processed by six DNA extraction methods to evaluate the assay inhibition effects. Briefly, bulk animal feed samples, including complete swine feed for various life stages and soybean meal (Table 3), were obtained from retail stores or feed mills and stored at room temperature. On the day of analysis, 25-g samples were aseptically weighed in sterile filter bags with 0.33 mm pore size (Whirl-Pak, Nasco Sampling, Pleasant Prairie, WI, USA) and suspended in 225 mL of phosphate-buffered saline (PBS; Thermo Fisher Scientific). After hand-massaging for 5 min, six sets of 20-mL aliquots from the filtered side of the bag were transferred to 50-mL Falcon tubes. The tubes were centrifuged at 900 × *g* for 3 min at 4 °C to remove large feed particles, and the supernatants were transferred to new tubes and centrifuged again at maximum speed (with Eppendorf 5430R rotor, Enfield, CT, USA) at 4 °C for 20 min. The pellets were stored at −20 °C before DNA extraction.

**Table 3:** Animal feed products evaluated by DNA extraction kits.

Sample ID	Product	Top 5 ingredients
1	Sow pig complete feed	Ground Corn, wheat middlings, distillers dried grains with solubles, corn germ meal, dehulled soybean meal
2	Organic mini pig adult feed	Barley, soybean meal, wheat middlings, oats, dehydrated alfalfa meal
3	Mature maintenance mini pig feed	Ground corn, wheat middlings, ground soybean hulls, oat hulls, ground oats
4	Mini pig active adult feed	Ground corn, wheat middlings, dehulled soybean meal, ground oats, dehydrated alfalfa meal
5	Sow and pig feed complete balanced nutrition for all life stages of pigs feed	Ground corn, wheat middlings, distillers dried grains with solubles, corn germ meal, dehulled soybean meal
6	Grower-finisher feed	Ground corn, wheat flour, dehulled soybean meal, cane molasses, soybean oil
7	Balanced hog nutrition feed	Ground corn, wheat middlings, dehulled soybean meal, Wheat Red dog, corn distillers dried grains with solubles
8	Soybean meal (7-1-2)	Soybean meal: Crude protein 46.0%; crude fat 0.5%; crude fiber 3.5%

The supernatant samples were prepared following the WOAH’s *Terrestrial Manual* sub-Chapter 3.9.1—African swine fever (infection with the African swine fever virus), Section 1.3—Detection of virus genome by the polymerase chain reaction (22). Briefly, 25-g samples were aseptically weighed in sterile filter bags and suspended in 100 mL of PBS. After mixing, six sets of 1-mL aliquots (representing 250-mg test portion) from the filtered side of the bag were transferred to individual 1.5-mL Eppendorf tubes. The tubes were centrifuged at 12,000 × *g* for 5 min. The clarified supernatants were stored at −20 °C before DNA extraction.

Six DNA extraction methods were evaluated. These include five commercial kits, ChargeSwitch, MagMAX CORE Nucleic Acid Purification Kit (manual extraction) (“MagMAX CORE” in short; Thermo Fisher Scientific), PrepMan Ultra Sample Preparation Reagent (“PrepMan”; Thermo Fisher Scientific), QIAamp Viral RNA Mini Kit (“QIAamp”; Qiagen, Germantown, MD, USA), and ZymoBIOMICS DNA Miniprep Kit (“ZymoBIOMICS”; Zymo Research, Irvine, CA, USA), and a crude extraction by boil prep. All DNA extraction protocols were performed using 300 μL input volume (except 140 μL for QIAamp and 1 mL for PrepMan and boil prep) following the manufacturers’ instructions. The complex workflow was used for the MagMax CORE manual extraction. Briefly, 300 μL of resuspended pellet in PBS or thawed supernatant was mixed with 450 μL of lysis buffer. After vortexing vigorously for 3 min, the mixture was centrifuged at 15,000 × *g* for 2 min to clarify the sample lysate. Aliquot (600 μL) of the supernatant was mixed with 30 μL of Bead Mix (20 μL of magnetic beads and 10 μL of proteinase K), vortexted for 1 min, and then centrifuged at 500 × *g* for 10 s. This process was followed similarly with Binding Solution (350 μL), Wash 1 solution (500 μL), and Wash 2 Solution (500 μL). After leaving tubes open on the magnetic stand to dry for 5 min, Elution Solution (90 μL) was added and the mixture was vortexed for 3 min, and then centrifuged at 500 × *g* for 10 s. After leaving the tubes on a magnetic stand for 3 min, the eluate was transferred to a fresh microcentrifuge tube and stored at −20 °C until use. For ZymoBIOMICS, bead-beading was performed on a Vortex Genie 2 with an adapter at full speed for 20 min. For PrepMan and boil prep, pellet samples were centrifuged at the maximum speed (~21,130 × *g*) for 4 min, and supernatant samples (1 mL) were centrifuged at the maximum speed for 2 min. After centrifugation, both types of pellets were resuspended in 100 μL of PrepMan (140 μL PBS for boil prep) and incubated at 100 °C for 10 min, followed by cooling to room temperature and centrifuged at 16,000 × *g* for 2 min to clear the supernatants, which were transferred to a fresh microcentrifuge tube and stored at −20 °C until use.

All sample DNA extracts were quantified using Quant-iT Broad-Range dsDNA Assay Kit on a Qubit fluorometer (Thermo Fisher Scientific). Aliquots (5 μL) of extracts were added to LAMP and real-time PCR reaction mixes along with 2 × 10^4^ copies of Quantitative Synthetic ASFV DNA (ATCC VR-3283SD) to evaluate any inhibitory effects in comparison with the baseline (molecular-grade water).

### 2.7 Animal feed evaluation with ASFV BA71V stock

The procedure followed the supernatant sample preparation above. Briefly, bulk complete swine feed was obtained from LabDiet (St. Louis, MO, USA), and a 100-g aliquot was spiked with ASFV BA71V stock at ~ca. 10^5.1^ TCID_50_/g. From the spiked sample, three 25-g test portions were suspended in 100 mL of PBS. The supernatants (50 mL) from each replicate were transferred to conical tubes and centrifuged at 12,000 × *g* for 5 min to pellet feed debris. The clarified supernatant was aliquoted (500 μL) and used for DNA extraction by ChargeSwitch and ZymoBIOMICS following the manufacturer’s instructions. The sample DNA extracts (5 μL) were subjected to LAMP and real-time PCR in three independent repeats.

### 2.8 Data analysis

Means and standard deviations of time-to-positive results (T_p_ for LAMP run on Genie II, C_T_ for LAMP run on 7500, and C_T_ for real-time PCR) were compared using the analysis of variance with Excel (Microsoft 365; Microsoft, Redmond, WA, USA). For sensitivity, LODs were presented as the lowest numbers of ASFV genome copies (or ASFV BA71V’s TCID_50_/mL levels or other ASFV isolates’ HAD_50_/mL levels) that could be detected by the assays. For specificity, inclusivity and exclusivity were presented as the percentage of detection. The animal feed inhibition effect was expressed as the delta T_p_ (dT_p_; min) or delta C_T_ (dC_T_; number of cycles) and delta T_a_ (dT_a_; °C) between the samples and controls. Comparisons among assays, kits, supernatants/pellets, and animal feed samples were conducted using the analysis of variance (ANOVA), followed by Kruskal–Wallis test or Tukey’s honestly significant difference (HSD) post-hoc test or post-hoc Dunn test using R version 2023.12.1 (47). Visualization of the dT_p_, dC_T_, and dT_a_ values was done with R using the ggplot2 package. The performance of the assays and kits in animal feed with ASFV BA71V stock was expressed as the probability of detection (POD) and rapidity (differences in T_p_/C_T_ values).

## 3 Results

### 3.1 Assay sensitivity

**Figure 1:**
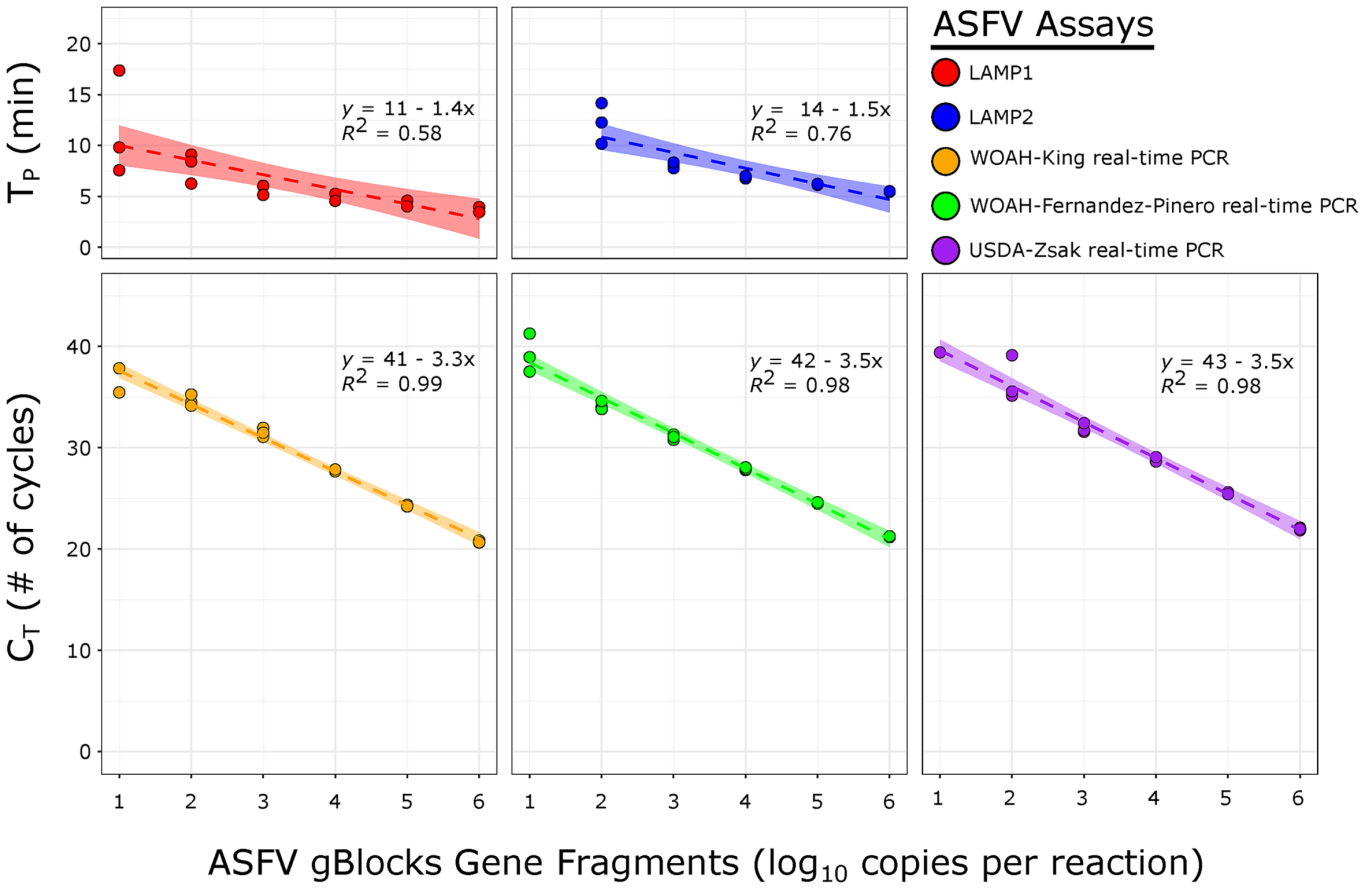
Standard curves generated using gBlocks Gene Fragments GEO 1/2007 (GEO 1/2007-P1192R in the case of LAMP2) by two LAMP assays—LAMP1 and LAMP2 (top panel) and three real-time PCR assays—WOAH-King, WOAH-Fernandez-Pinero, and USDA-Zsak (bottom panel).

Table 4 summarizes the LAMP and real-time PCR assay LODs evaluated using 10-fold serial dilutions of ASFV synthetic DNAs or BA71V stock in three independently run assays. Overall, LODs varied by sample type and assay type. Using synthetic gBlocks Gene Fragments for the ASFV Georgia strain, LAMP1 reached an LOD of 10 copies/reaction compared to 100 copies/reaction for LAMP2. For templates ranging from 10^6^ to 10^2^ copies/reaction, corresponding average T_p_ values fell from 3.8 ± 0.3 to 7.9 ± 1.5 min for LAMP1 and from 5.5 ± 0.0 to 12.2 ± 2.0 min for LAMP2, with standard curve coefficients of determination (*R^2^*) of 0.58 and 0.76, respectively (Figure 1). The average T_p_ for LAMP1 at the 10^1^ copies/reaction level was 11.6 ± 5.1 min. All three real-time PCR assays detected 10 copies/reaction with the gBlocks Gene Fragment, though not all three repeats were positive at this level (two positives for WOAH-King and one positive for USDA-Zsak). For templates ranging from 10^6^ to 10^1^ copies/reaction, corresponding average C_T_ values fell from 20.7 ± 0.1 to 36.7 ± 1.7 cycles (WOAH-King), 21.2 ± 0.0 to 39.2 ± 1.9 cycles (WOAH-Fernandez-Pinero), and 22.0 ± 0.1 to 39.4 cycles (USDA-Zsak), with all having an *R^2^* ≥ 0.98 (Figure 1).

**Table 4:** Assay sensitivity evaluated using ASFV synthetic DNAs and BA7IV virus stock for LAMP1 and the USDA-Zsak or USDA-APHIS real-time PCR in three independently run assays.

Sample type	Detection limit (copies/reaction for ASFV synthetic DNAs and TCID_50_/for ASFV mLBA7IV)				
	LAMP1	LAMP2	USDA-Zsak or USDA-APHIS real-time PCR^c^	WOAH-King	WOAH-Fernandez-Pinero
gBlocks Gene Fragment (GEO 1/2007 or GEO 1/2007-P1192R for LAMP2)	10	100	10^a^	10^b^	10
Quantitative synthetic ASFV DNA (ATCC VR-3283SD)	8.8	NA	0.88^a^	0.88^b^	0.88^a^
BA71V virus stock	~10^b^	NA	~10	NA	NA

a One out of three repeats was positive for this detection limit.

b Two out of three repeats were positive for this detection limit.

c ASFV synthetic DNAs were evaluated by the USDA-Zsak real-time PCR, whereas ASFV BA71V stock was evaluated by the USDA-APHIS real-time PCR.

Using Quantitative Synthetic ASFV DNA ATCC VR-3283SD, LAMP1 and the three real-time PCR assays consistently detected 8.8 copies/reaction, whereas the latter also detected 0.88 copy/reaction in one to two repeats (Table 4). For templates ranging from 8.8 × 10^5^ to 8.8 copies/reaction, corresponding average T_p_ values for LAMP1 fell from 4.4 ± 0.1 to 9.2 ± 2.1 min with an *R^2^* of 0.77, and the corresponding average C_T_ values fell from 20.2 ± 0.2 to 35.9 ± 0.0 cycles (USDA-Zsak), 20.8 ± 0.3 to 38.1 ± 0.2 cycles (WOAH-King), and from 20.8 ± 0.3 to 36.9 ± 0.4 cycles (WOAH-Fernandez-Pinera), all with *R^2^* ≥ 0.99 (data now shown). In one repeat, both the USDA-Zsak real-time PCR and the WOAH-Fernandez-Pinera real-time PCR detected the 0.88 copies/reaction level with a C_T_ of 39.9 cycles, and in two repeats, the WHO-King real-time PCR also detected this level with an average C_T_ of 39.4 ± 0.7 cycles.

For the Vero cell-adapted ASFV BA71V stock, both LAMP1 and the USDA-APHIS real-time PCR detected ca. 10^1^ TCID_50_/mL, though the former had one repeat failed detection at this level (Table 4). For LAMP1 run on 7500, average C_T_ values for ASFV BA71V stocks ranging from 10^7^ to 10^2^ TCID_50_/mL fell from 3.7 ± 0.0 to 10.1 ± 0.7 cycles (1 min per cycle) with an *R^2^* of 0.87 (data not shown). Average C_T_ values for the USDA-APHIS real-time PCR for the same series fell from 20.6 ± 0.5 to 36.2 ± 0.3 cycles (~40 s per cycle) with an *R^2^* of 1 (data not shown).

### 3.2 Assay specificity

Among the 27 ASFV synthetic DNAs (26 gBlocks Gene Fragments and 1 certified reference), representing the p72 gene of various genotypes, one (R35) belonging to genotype V was not detected by LAMP1 (96.3% inclusivity), whereas the WOAH-King real-time PCR (92.6% inclusivity) failed to detect two (RSA_2_2004 and Zaire, both genotype III), and the other two real-time PCR assays had 100% inclusivity (Table 1). Testing R35 at concentrations higher than that used for inclusivity (10^4^ copies/reaction) resulted in detection by LAMP1 starting at 10^6^ copies/reaction with a T_p_ of 11.9 min. The T_p_ values for LAMP1 averaged 6.3 ± 1.3 min (range, 5.0–9.0 min). The three real-time PCR assays had average C_T_ values for WOAH-King of 25.1 ± 4.0 cycles (range, 22.6–39.9 cycles), for WOAH-Fernandez-Pinero of 26.6 ± 1.5 cycles (range, 24.3–29.3 cycles), and for USDA-Zsak of 27.9 ± 3.2 cycles (range, 25.1–35.5 cycles), respectively. One isolate (RSA_2_2008, genotype III) had the highest T_p_/C_T_ values by LAMP1 (9.0 min) and the WOAH-King real-time PCR (39.9 cycles), and a high C_T_ by the USDA-Zsak real-time PCR (34.7 cycles). Another isolate (SPEC_57, genotype III) had C_T_ values > 35 cycles for the WOAH-King and the USDA-Zsak real-time PCRs, as well as a high LAMP1 T_p_ of 8.3 min. The two isolates (RSA_2_2004 and Zaire, both genotype III) not detected by the WOAH-King real-time PCR had C_T_ values > 35 cycles by the USDA-Zsak real-time PCR and a high LAMP1 T_p_ > 8 min. Notably, all C_T_ values for the WOAH-Fernandez-Pinero real-time PCR were < 30 cycles (Table 1).

Among the nine ASFV isolates for inclusivity evaluation, all were detected by LAMP1 and the USDA-APHIS real-time PCR, suggesting 100% inclusivity. The LAMP1 T_p_-averaged 8.6 ± 1.8 min (range, 5.0–11.1 min), whereas the USDA-APHIS real-time PCR C_T_ averaged 28.8 ± 1.1 cycles (range, 26.6–30.0 cycles). None of the non-ASFV porcine viruses (*n* = 13) were detected by LAMP1 or the USDA-APHIS real-time PCR, suggesting 100% exclusivity (Table 1).

### 3.3 Effect of DNA extraction method on animal feed inhibition

**Figure 2:**
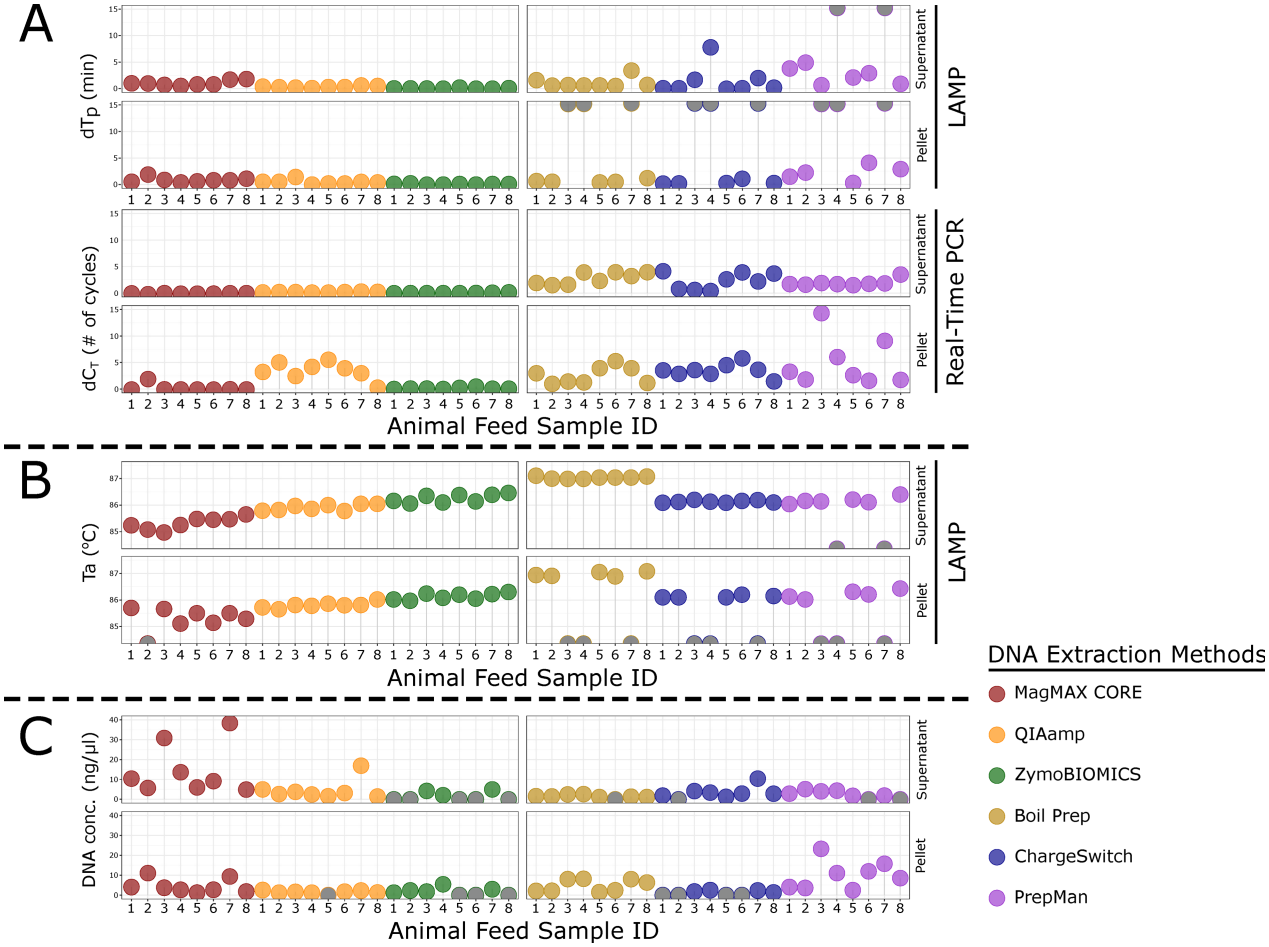
Lollipop charts showing the effects of six DNA extraction methods on LAMP1 and the USDA-Zsak real-time PCR performance using eight representative types of animal feed products (supernatants and pellets) on the differences in LAMP1 T_p_ (dT_p_; min) or USDA-Zsak real-time PCR C_T_ (dC_T_; number of cycles) values **(A)**, LAMP1 T_a_ (°C) values **(B)**, and DNA concentrations by animal feed sample and kit (supernatants and pellets) **(C)**. The sample IDs are the same as those shown in Table 3. Grey dots show samples with no detection by LAMP1.

The effect of six DNA extraction methods was evaluated using supernatant and pellet samples generated from eight types of animal feed products and analyzed by LAMP1 and the USDA-Zsak real-time PCR (Figure 2). Though there were no statistical differences in T_p_/C_T_ or T_a_ between supernatant and pellet, or among the eight animal feed products (*p* > 0.05), both assay type and DNA extraction method and their combinations significantly impacted T_p_/C_T_ (*p* < 0.001), and T_a_ also differed significantly by DNA extraction methods (*p* < 0.001).

Among supernatant samples tested by LAMP1, the lowest inhibition was seen with ZymoBIOMICS, where dT_p_ averaged 0.04 ± 0.1 min, whereas the highest inhibition was seen with PrepMan, where dT_p_ averaged 2.5 ± 1.7 min (*p* < 0.001) (Figure 2A). Two supernatant samples extracted by PrepMan, namely sample 4 (Mini pig active adult feed) and sample 7 (Balanced hog nutrition feed), were undetectable by LAMP1. Among supernatant samples tested by the USDA-Zsak real-time PCR, the lowest inhibition was seen with MagMAX CORE, where dC_T_ averaged −0.07 ± 0.1 cycles, and the highest inhibition was with boil prep, where dC_T_ averaged 2.8 ± 1.1 cycles (*p* < 0.001) (Figure 2A).

Among pellet samples tested by LAMP1, the lowest inhibition was seen with ZymoBIOMICS, where dT_p_ averaged 0.07 ± 0.1 min, and the highest with PrepMan, where dT_p_ averaged 2.2 ± 1.4 min (*p* < 0.001) (Figure 2A). Three pellet samples, namely sample 3 (Mature maintenance mini pig feed), sample 4 (Mini pig active adult feed), and sample 7 (Balanced hog nutrition feed) extracted by boil prep, ChargeSwitch, and PrepMan, were undetectable by LAMP1. Among pellet samples tested by the USDA-Zsak real-time PCR, similarly, the lowest inhibition was seen with ZymoBIOMICS, where dC_T_ averaged 0.1 ± 0.1 cycles, and the highest inhibition was seen with PrepMan, where dC_T_ averaged 5.0 ± 4.6 cycles (*p* < 0.001) (Figure 2A).

Interestingly, the T_a_ of LAMP1 varied greatly based on DNA extraction methods, with the most obvious difference seen in samples extracted by boil prep, averaging 1 °C higher than those in samples extracted by MagMAX CORE (*p* < 0.001) (Figure 2B). No T_m_ info was available for the USDA-Zsak real-time PCR, as it was a probe-based assay. Notably, the amount of DNA extracted from the samples varied greatly, ranging from an average concentration of 1.7 ± 0.6 ng/μL in boil-prepped supernatants to 14.9 ± 12.6 ng/μL in MagMAX CORE-extracted supernatants (Figure 2C). There were eight incidences for ZymoBIOMICS, five for ChargeSwitch, two for PrepMan, and one each for QIAamp and boil prep that the DNA extracts were too low to be quantified by the Qubit BR kit (Figure 2C).

### 3.4 Rapid detection of ASFV in spiked animal feed

**Figure 3:**
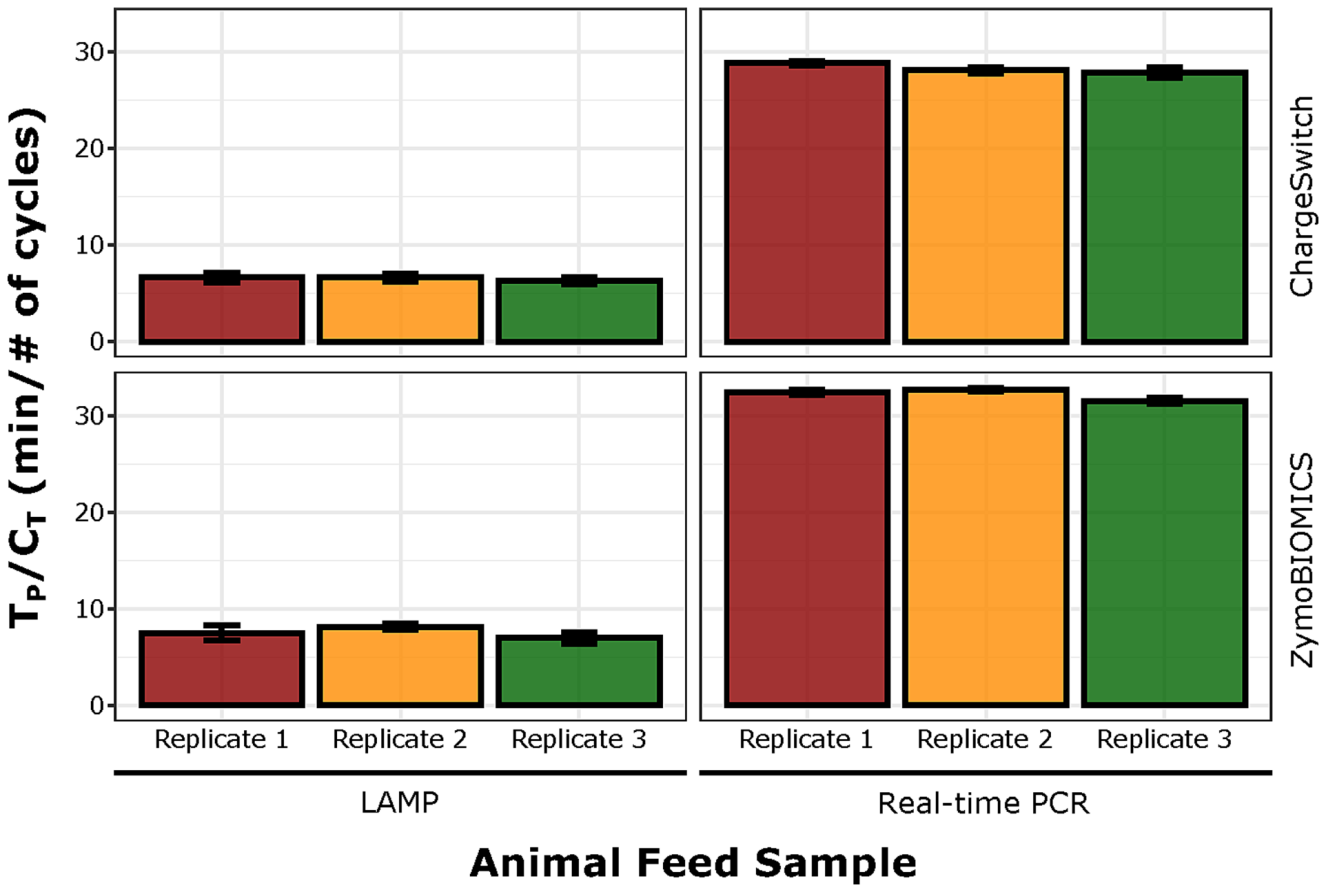
Comparative detection by LAMP1 and the USDA-APHIS real-time PCR in complete swine feed with the infectious ASFV BA71V virus stock spiked at 10^5.1^ TCID_50_/g and DNA extracted by ChargeSwitch (top panel) and ZymoBIOMICS (bottom panel).

All kit/assay combinations yield 100% detection when the ASFV BA71V virus stock was inoculated in complete swine feed at 10^5.1^TCID_50_/g. Using DNA extracted with ChargeSwitch, the newly developed LAMP1 assay reliably detected the virus within 7 min in contrast to at least 20 min by the real-time PCR (29 cycles) (Figure 3). For samples extracted with ZymoBIOMICS, LAMP1 reaction time was extended by approximately 1 min and the real-time PCR extended by approximately 4 cycles to reach positive detection (Figure 3).

## 4 Discussion

This ASFV feed detection method development and evaluation work highlights the rapidity, specificity, sensitivity, and utility of the newly developed p72 gene-based ASFV LAMP assay (i.e., LAMP1) in animal feed. Compared to the first published, field-verified LAMP assay (LAMP2) targeting the topoisomerase II gene (41, 42), LAMP1 was faster (3.8 min vs. 5.5 min for ~10^6^ copies of gBlocks Gene Fragments per reaction) with 10-fold higher sensitivity (10 copies versus 100 copies). Extensive specificity evaluation using 26 synthesized p72 gene fragments representing various ASFV genotypes showed good inclusivity, comparable to the two real-time PCR assays recommended by the WOAH (22, 28, 29), and another real-time PCR developed by USDA-ARS and currently used by USDA-APHIS National Veterinary Services Laboratories (NVSL) (30, 48). Both inclusivity and exclusivity were confirmed with various swine viruses, along with sensitivity, which varied by ASFV strain and assay type. Further, the comprehensive evaluation of DNA extraction kits in a variety of animal feed sample types clearly demonstrated varied inhibitory effects in these matrices and the kit performance. This work contributes toward a rapid and reliable screening tool for ASFV feed surveillance, outbreak response, and recovery efforts to safeguard the nation’s animal feed supply.

Owing to its simplicity, versatility, and tolerance to assay inhibitors, LAMP has gained global recognition for its potential in field application (40). In 2010, United Kingdom scientists described the first ASFV LAMP assay (i.e., LAMP2) using three signal detection formats (agarose gel, real-time PCR machine, and lateral flow device) and its application in blood and tissue samples (41). Using ASFV Malta/78 DNA, the assay run was 25.3 ± 1.2 min without the loop primers and 11.3 ± 0.6 min with them. The analytical sensitivity (330 genome copies) was good and inclusivity was 100% using 38 ASFV isolates of various genotypes. In diagnostic samples, LAMP and real-time PCR each failed only in two incidences compared to the gold standard virus isolation (41). In-field verification of LAMP2 was done during an outbreak in Timor-Leste, where DNA extraction was removed and an IAC was adopted (42). Similar to the study presented herein, OptiGene Isothermal Mastermix 004 and a portable fluorometer (a smaller Genie III vs. Genie II) were used. A colorimetric LAMP was also evaluated, and a portable real-time PCR was compared. The authors reported a substantial level of agreement between LAMP and real-time PCR in both serum and oral/rectal swabs (42). Notably, despite ASFV LAMP having lower sensitivity than ASFV real-time PCR, both studies (41, 42) reported clinical samples positive by LAMP but negative by real-time PCR, likely attributable to better LAMP tolerance to inhibitors in the samples. In Timor-Leste, LAMP2 was then used in a large-scale field prevalence survey and proof of freedom survey with limited laboratory facilities due to LAMP’s proven robustness, high specificity, and sensitivity (49).

Since 2020, multiple LAMP assays targeting various ASFV genes have been reported, e.g., p10 (50), *C962R* (51), *9GL (B119L)* (52), and *B646L* (53–62). Diverse LAMP assay formats were used, including colorimetric (54, 61, 63), lateral flow dipstick (59), carbon nanodot-based biosensor (57), CRISPR/Cas12a-mediated (58, 60), fluorescence (53), and microfluidic (62), illustrating LAMP versatility. LAMP1 LODs in the present study (8.8–10 genome copies) fell within the range (1–100 genome copies) observed in those studies when synthetic DNAs were used. For the ASFV BA71V stock, both LAMP1 and the USDA-APHIS real-time PCR had an LOD of ca. 10^1^ TCID_50_/mL, confirming that the LOD established with ASFV synthetic DNAs was valid. For the inclusivity testing, delayed assays were observed, particularly for strains belonging to genotype III (Table 1). This reflected sequence variations in the p72 gene region targeted by the LAMP1 primers and real-time PCR primers/probes. Even after incorporating degenerative primers to account for such variations, genotype- and strain-dependent LODs (100-fold) were still observed (53). Nonetheless, the near 100% inclusivity and exclusivity of LAMP1 and three real-time PCR assays agreed with earlier reports testing a limited number of strains in both panels (53). In the present study, the use of synthesized p72 gBlocks Gene Fragments of various ASFV strains representing diverse genotypes effectively solved the issue of strain availability for inclusivity testing. Notably, the *R^2^* values (linearity quantification) for LAMP1 and LAMP2 were poorer compared to all three real-time PCR assays, which was attributable to the highly speedy nature of LAMP amplification (quasi-exponential) versus that of real-time PCR (exponential) (38).

LAMP suitability for ASFV diagnostics in a range of clinical sample types, including whole blood, serum, tissue, spleen, liver, tonsil, oral fluid, and meat, has been adequately demonstrated (50, 53, 54, 57). Due to inherent challenges with ASFV detection in animal feed, particularly ample assay inhibitors, for example, organic matters (64) and low viral loads, the wide applicability of rapid molecular methods such as LAMP and real-time PCR in these commodities has not been demonstrated. For inhibitor removal and viral concentration from animal feed, to enable quality DNA for rapid ASFV detection, DNA extraction plays a critical role, but published studies are scarce. A recent report by the Diel group (65) optimized the protocol of MagMAX CORE Nucleic Acid Purification Kit (i.e., MagMAX CORE) in animal feed ingredients and complete swine feed and evaluated the detection of ASFV and Senecavirus A (SVA, an RNA virus) by real-time PCR [VetAlert African Swine Fever Virus DNA Test Kit (Tetracore, Rockville, MD, USA) and EZ-SVA RT-PCR (Tetracore), respectively]. An LOD of 922 copies of a pUC57 plasmid containing the ASFV p72 gene was shown in complete swine feed, distiller’s dried grains with soluble (DDGS), lysine, and vitamin D, while whole corn and soybean meal presented LODs of 92 copies and 9.2 copies, respectively. A follow-up report (66) showed that the MagMAX CORE kit outperformed two other kits [IndiMag Pathogen Kit (Indical BioScience, Orlando, FL, USA) and MagMAX Viral/Pathogen II Nucleic Acid Isolation Kit (MVP II; Thermo Fisher Scientific)] in extracting viral RNA [porcine reproductive and respiratory syndrome virus (PRRSV), SVA, or porcine epidemic diarrhea virus (PEDV)] from animal feed ingredients and feed mill environmental samples, evidenced by lower C_T_ and higher sensitivity. Both reports clearly demonstrated the effects of DNA extraction method on ASFV detection in animal feed.

In the present study, animal feed supernatants and pellets were subjected to DNA extraction by six methods, followed by analysis with LAMP1 and the USDA-Zsak real-time PCR. The use of supernatant agreed with the Diel group’s optimized MagMAX CORE protocol (65, 66) as well as that specified in the WOAH’s *Terrestrial Manual* (22). The use of pellets was to mimic traditional virus processing, where ultracentrifugation was used to concentrate viruses in the sample prior to nucleic acid extraction (67). Notably, the feed sample to PBS ratio varied from 1:4 in Diel’s study supernatant (5 g to 15 mL PBS) to 1:5 in WOAH supernatant (25 g to 100 mL PBS) to 1:10 in pellet samples in the present study (25 g to 225 mL PBS). Using the same 300 μL input volume for most in the kit comparison portion of this study, regardless of supernatant or pellet, the ZymoBIOMICS kit had the least inhibition for LAMP1, whereas the MagMAX CORE kit performed the best for the USDA-Zsak real-time PCR with the ZymoBIOMICS kit being a close second (Figure 2). Crude DNA extraction by boil prep and a quick heating protocol by PrepMan did not perform as well, indicating relatively low efficiencies at removing assay inhibitors from the animal feed samples.

We then applied ZymoBIOMICS and ChargeSwitch in the animal feed spiking trial since the latter was currently used at the FDA’s field laboratories and several state laboratories for screening animal feed for prohibited animal species (68). The consistent detection of the ASFV BA71V virus stock spiked at 10^5.1^ TCID_50_/g in complete swine feed by both LAMP1 and the USDA-APHIS real-time PCR demonstrated the feasibility of these detection strategies (kit and assay combinations), particularly considering the reported minimum infectious dose of ASFV in feed was 10^4^ TCID_50_ (median, 10^6.8^ TCID_50_) (69). Upon further validation, this newly developed p72 gene-based LAMP assay may be adopted as a viable option for use in routine ASFV feed surveillance, with the distinct advantages over real-time PCR in terms of speed and portability in outbreak situations. We acknowledge that molecular assays, including LAMP and real-time PCR, are inherently lacking the ability to unequivocally differentiate infectious from non-infectious viruses; thus, further confirmation using traditional virus isolation is warranted in any samples that screen positive. Nonetheless, this work establishes the initial framework to greatly expand our toolbox for enhanced ASFV detection to safeguard the nation’s animal feed supply.

## 5 Conclusion

Given the inherent risks of ASFV accidental introduction into the United States through imported animal feed from ASF enzootic regions, the transmission after introduction via contaminated feed, combined with the high stability of ASFV in feed, rapid and early detection serves as one of the first steps among many biosecurity measures to establish effective prevention and control strategies for this often deadly, transboundary animal disease. The novel p72 gene-based ASFV LAMP assay developed and evaluated in the present study was rapid, specific, sensitive, and feasible in animal feed. Further validation of this screening tool will facilitate its adoption by the FDA’s field laboratories for enhanced ASFV surveillance in animal feed and prevention and outbreak response efforts.

## Data Availability

The original contributions presented in the study are included in the article and further inquiries can be directed to the corresponding author.
